# Proliferation, migration, and differentiation of circulating ILC precursors in HBV infection-associated fibrosis

**DOI:** 10.1186/s40001-025-03803-w

**Published:** 2026-01-05

**Authors:** Qin Zhu, Fang-yuan Chen, Shi-qin Li, Cheng-zhao Weng, Si-qi Wang, Lin-lin Zheng, Yong-yu Yang, Li Xie, Jian Wu, Wei Jiang

**Affiliations:** 1 Department of Gastroenterology and Hepatology, Zhongshan Hospital (Xiamen), Fudan University, Xiamen, 361015 China; 2 Department of Gastroenterology and Hepatology, Zhongshan Hospital of Fudan University, 180 Fenglin Road, Shanghai, 200032 China; 3 Shanghai Institute of Liver Diseases, Fudan University Shanghai Medical College, Shanghai, 200032 China; 4 Shanghai Institute of Infectious Disease and Biosecurity, Fudan University, Shanghai, 200032 China; 5 Department of Allergy, Zhongshan Hospital, Fudan University, Shanghai, 200032 China; 6 Department of Infectious Diseases, Zhongshan Hospital, Fudan University, Shanghai, 200032 China; 7 Department of Medical Microbiology and Parasitology, MOE/NHC/CAMS Key Laboratory of Medical Molecular Virology, School of Basic Medical Sciences, Fudan University Shanghai Medical College, Shanghai, 200032 China

**Keywords:** Chronic hepatitis B infection, Circulating innate lymphoid cell precursors, Innate lymphoid cells, Notch signaling pathway, Interleukin-23

## Abstract

**Background:**

Increased studies indicate that innate lymphoid cells (ILCs) are involved in inflammatory and immune responses in chronic liver injury. However, recruitment and maturation of their subsets during hepatic fibrosis post-HBV infection are unknown. The present study aims to explore the potential impact of ILC precursors (ILCPs) on the modulation of HBV infection-associated fibrosis.

**Methods:**

Peripheral blood mononuclear cells (PBMCs) from healthy controls (HCs), chronic hepatitis B infected (CHB), and cirrhotic patients and lymphoid and splenic mononuclear cells from HBV-transgenic mice were isolated to determine the richness of ILC precursors (ILCPs) and ILC subsets by flow cytometry.

**Results:**

The richness of ILCPs and ILC3 subsets was significantly increased in PBMCs of patients with chronic hepatitis B infection and in lymph nodes and spleens of HBV-transgenic mice with carbon tetrachloride treatment. Importantly, among these ILCPs, the percentage of CD62L and CXCR6-expressing ILCPs were proportionally enriched. IL-23 may contribute to CD62L^+^ and CXCR6^+^ ILCP recruitment and differentiation into ILC3 subsets; whereas a Notch signaling inhibitor DAPT exerted inhibitory effects on them.

**Conclusion:**

The microenvironment of HBV infection-associated fibrosis enhances the proliferation, migration, and differentiation of peripheral ILCPs and IIL-23, and Notch signaling pathways may play significant roles in these processes.

**Supplementary Information:**

The online version contains supplementary material available at 10.1186/s40001-025-03803-w.

## Introduction

Hepatitis B virus (HBV) infection remains a formidable global health burden, with estimated 296 million individuals living with chronic infection, which significantly elevates the risk for liver fibrosis, cirrhosis, and hepatocellular carcinoma [1, 2]. The progression of HBV infection-associated fibrosis is a multifaceted process characterized by persistent hepatic inflammation, recurrent cycles of hepatocyte damage and repair, and the sustained activation of hepatic stellate cells (HSCs) [3, 4]. This pathological landscape is sculpted by a complex interplay between enduring viral antigens and a dysregulated host immune response [5].

Among the immune cell populations involved, innate lymphoid cells (ILCs) have surfaced as pivotal regulators of tissue homeostasis, inflammation, and fibrotic processes [6, 7]. ILCs are categorized into three groups, including ILC1s, ILC2s, and ILC3s, based on their developmental reliance on specific transcription factors and their functional cytokine profiles, which mirror those of T helper 1 (Th1), Th2, and Th17 cells, respectively [8]. They act as rapid responders to environmental changes and are crucial in the initial phases of immune regulation and tissue repair [9]. Within the liver, ILCs are increasingly implicated in a variety of pathologies, including viral infection, cancer development, and fibrogenesis [10–12].

Particular focus has been placed on ILC3s, which are notable for their production of IL-17A and IL-22, in the context of chronic liver disease [13, 14]. Our previous research, consistent with other studies, has demonstrated that ILC3s expand in number and contribute to liver fibrosis progression by activating HSCs through cytokine-mediated mechanisms [15, 16]. However, while the effector functions of mature, tissue-resident ILC3s within the liver are becoming clearer, the ontological source and regulatory mechanisms governing these cells during chronic HBV infection remain inadequately understood.

Circulating ILC precursors (ILCPs) have been identified as a population with the capacity to differentiate into all helper ILC subsets upon migration into peripheral tissues [17]. These precursors express critical homing receptors, including CD62L (L-selectin), which facilitates their entry into secondary lymphoid organs via high endothelial venules [18], and CXCR6, which is essential for their retention and recruitment to peripheral sites such as the liver [19]. The differentiation and migratory patterns of ILCPs are believed to be directed by local tissue-derived signals. The cytokine IL-23, frequently elevated in chronic inflammatory states, and the evolutionarily conserved Notch signaling pathway, have been strongly implicated in ILC development, functional plasticity, and effector responses [20–22].

Notwithstanding these advancements, it remains unknown whether circulating ILCPs are dysregulated during the progression of HBV infection-associated fibrosis, and if so, which molecular mechanisms govern their recruitment and differentiation. This study aims to address this knowledge gap by characterizing the phenotypic and functional dynamics of circulating ILCPs in patients with chronic HBV infection and associated fibrosis stages, supported by a complementary murine model. We further elucidate the potential contributory roles of the IL-23 cytokine and the Notch signaling pathway in modulating these processes. Our findings offer novel insights into the immunopathology of HBV-associated liver disease by underscoring the circulation and fate determination of ILC precursors as a previously underappreciated axis in fibrotic progression.

## Materials and methods

### Subjects

Patients with HBV e antigen (HBeAg)-positive chronic hepatitis B (CHB, *n* = 18) or HBV infection-associated liver cirrhosis (LC, *n* = 22) were enrolled between September 2022 and June 2023 at Zhongshan Hospital of Fudan University (Shanghai, China). In patients with CHB and liver cirrhosis, HBV-DNA levels were below 50 IU/mL after 100 months of entecavir monotherapy, without any complications. Healthy controls (HCs, *n* = 12) were recruited from volunteers for routine health examinations in Zhongshan Hospital. Patients with CHB were diagnosed as previously reported [23]. The patient demography and clinical characteristics are summarized in Table 1. LC was confirmed by explicit morphological criteria with ultrasound, fibroscan, computed tomography, magnetic resonance imaging, or the presence of cirrhotic complications (variceal bleeding or ascites). The exclusion criteria include the positivity of other viral infection, active alcohol abuse, autoimmune disease, fatty liver disease, and tumors. All participants gave informed consent before joining the study. They received a detailed information sheet outlining the study's goals, procedures, potential risks and benefits, and their right to withdraw at any time. Written consent was obtained through signed forms. The study followed the World Medical Association Declaration of Helsinki and was approved by Zhongshan Hospital's Research Ethics Committee [No. B2017-192(3)].

**Table 1:** Clinical characteristics of enrolled subjects

Group	HCs	CHB	LC	*P* value
Number of subjects	12	18	22	ns
Age (years)	50 ± 16.5	50 ± 11.44	56.45 ± 10.2	ns
Gender (M/F)	7/5	13/5	17/5	ns
Antiviral therapy (weeks)	0	466.5 ± 19.44	478.6 ± 22.21	ns
RBC (10E12/L)	4.557 ± 0.5451	5.957 ± 0.498	4.942 ± 0.789	ns
Hb (g/L)	136.7 ± 17.23	150.2 ± 12.54	147.8 ± 24.33	ns
WBC (10E9/L)	6.579 ± 1.373	5.78 ± 1.055	4.964 ± 1.498	ns
N%	57.26 ± 8.74	62.19 ± 6.56	62.17 ± 11.11	ns
PLT (10E9/L)	235.7 ± 45.59	209.2 ± 55.13	143.2 ± 68.59	0.0021
ALT (U/L)	23.64 ± 12.29	24.91 ± 21.62	27.82 ± 11.84	ns
AST (U/L)	20.80 ± 5.16	24.88 ± 9.39	24.77 ± 6.39	ns
GGT (U/L)	24 ± 11.37	23.63 ± 9.824	34.18 ± 22.48	ns
ALP (U/L)	62 ± 25.84	77.17 ± 26.16	83.05 ± 21.04	ns
CHE (U/L)	/	10,454 ± 3299	8147 ± 2180	0.0116
ALB (g/L)	42 ± 2.490	47.94 ± 2.209	48 ± 4.320	ns
TBIL (μmol/L)	9.822 ± 4.864	13.75 ± 6.056	15.5 ± 6.533	ns
DBIL (μmol/L)	2.589 ± 0.3093	3.117 ± 1.093	3.841 ± 2.157	ns
FBG (mmol/L)	5.622 ± 1.305	6.861 ± 6.035	5.945 ± 1.700	ns
Cholesterol (mmol/L)	4.725 ± 0.8001	4.497 ± 0.610	4.418 ± 0.88	ns
AFP (ng/mL)	3.603 ± 2.194	2.453 ± 1.573	1.991 ± 0.953	ns
PT (s)	11.52 ± 0.2182	11.87 ± 0.446	12.53 ± 1.038	ns
INR	0.9827 ± 0.5676	1.012 ± 0.045	1.060 ± 0.089	ns
HBV-DNA (IU/mL)	/	< 50	< 50	ns
Fibro Scan E (kPa)	/	5.831 ± 1.759	11.87 ± 6.155	0.0005

Data are shown as mean ± standard deviation (SD) with number of subjects. “ns” indicates not significant. *N%* neutrophil percentage, *PLT* platelet, *CHE* cholinesterase, *FBG* fasting blood glucose, *PT* prothrombin time, *INR* international normalized ratio, *Fibro Scan E* Fibro Scan examination

### Isolation and culture of human PBMCs

PMBCs were isolated from HCs, CHB, and cirrhotic patients using a Ficoll-Paque gradient (Serumwerk #00422, Bernburg, Germany) as previously described [15], and were stored in fetal bovine serum containing 10% DMSO at -80℃. To assess the effects of IL-23 and Notch receptors on ILCP expression, function, and differentiation, PBMCs from cirrhotic patients were resuscitated and were cultured in RPMI medium 1640 with the addition of 1.0 g/L glucose, 10% fetal bovine serum (FBS), penicillin, and streptomycin at 100 mg/mL at 37 ℃ in a humidified environment composed of 5% CO2 in the air. IL-2, IL-7, and IL-1β cytokines at 50 ng/mL were added in PMBCs to induce the differentiation of ILCs. Then, IL-23 (50 ng/mL), the Notch signaling inhibitor DAPT (N-[N-(3,5-difluorophenacetyl)-L-alanyl]-S-phenylglycine t-butyl ester; 20 μM), or an equal volume of PBS (control) was added to the cultures. After 3 days of induction, cells were collected for flow cytometric analysis.

### Design of mouse experiments

Experiments were approved by the ethical policies and procedures approved by the Animal Care Committee of Zhongshan Hospital, Fudan University (2023–152). Six-week male HBV-transgenic (Tg) mice on a C57BL/6NCrl background were purchased from Beijing Vitalstar Biotechnology Co., Ltd. (Beijing, China), containing terminally redundant, 1.28-fold length copies of the complete HBV genome, which were created based on the method of microinjection technology of fertilized egg [24]. These mice are widely used for HBV infection, immune abnormality, and antiviral therapy [25–27], although there is minimal fibrotic progression when they grow to adult age. Mice were maintained in a specific pathogen-free (SPF) facility with an environmental control system and were housed four per 36.5 × 28 × 11 cm cage with a 12-h light–dark cycle and free access to food and water. After 2 weeks of adjustable feeding, a total of 16 HBV-transgenic (Tg) mice were randomly assigned to two experimental groups: the HBV-Tg group (*n* = 8) and the HBV-Tg + CCl₄ group (*n* = 8) [15]. One mouse from each group was allocated for pilot experiments to optimize the flow cytometry gating strategy and antibody panel and was therefore not included in the final data analysis. Consequently, all results presented in this study are derived from 14 mice, with seven in each group (HBV-Tg, *n* = 7; HBV-Tg + CCl₄, *n* = 7). Mice were intraperitoneally injected with carbon tetrachloride (CCl_4_, dissolved in olive oil with a volume ratio of 40%, 2 μL/g body weight) or olive oil of equal volume twice a week for 8 weeks to potentiate fibrotic deposition in the liver. All mice were administered sodium pentobarbital (30 mg/kg) via intraperitoneal injection to induce deep anesthesia. Once confirmed to be fully anesthetized, cervical dislocation was performed. After euthanasia, liver tissues were isolated and fixed in 10% neutral buffered formalin for histological analysis. For the quantification of intrahepatic HBV-DNA, total DNA was extracted from mouse liver tissues using the TIANamp Genomic DNA Kit (Tiangen Biotech, #DP304, Beijing, China), according to the manufacturer's protocol. Serum HBV-DNA levels were measured using the DAAN Gene Real-Time Fluorescent PCR Kit for HBV-DNA (DAAN Gene, #DA0030–DA0032, Guangzhou, China), according to the manufacturer's instructions. Amplification and detection were performed on a Thermo Fisher Scientific ABI 7500 Real-Time PCR System. The assay has a dynamic linear range of 1 × 10^2^ to 1 × 10⁸ IU/mL, with a lower limit of quantification of 20 IU/mL. The spleens and lymph nodes were immediately isolated for flow cytometric analysis and detailed steps are described below.

### Histology and immunohistochemical staining

Liver tissue samples were preserved in formalin, embedded in paraffin, and dehydrated. Paraffin-embedded liver sections underwent H&E, Masson, and Sirius red staining to examine liver structure and evaluate fibrosis. An immunohistochemistry-based semi-quantitative analysis of hepatic fibrosis was performed using Image-J software in a blinded manner to estimate its severity. An immunohistochemical staining was performed as previously described [28].

### Isolation of mononuclear cells from mouse spleens and lymph nodes

Spleen and lymph nodes were removed and dissociated in 10% fetal bovine serum (FBS) RPMI 1640 medium. Mononuclear cells were collected by pressing spleen and lymph nodes through a plunger from a 5 ml syringe in the 70 m cell strainer. After transfer to a 15 ml centrifuge tube, the cellular suspension was centrifuged at 400 ×*g* for 10 min. Red blood cells in the spleen were lysed by 10X RBC Lysis Buffer (Peprotech-BioGems, 64,010–00-100, New Jersey, USA) diluted to 1X using double distilled water. Mononuclear cells obtained from spleen and mesenteric lymph nodes were washed twice by PBS and then used for flow cytometric analysis.

### Flow cytometric analysis

Dead cells of both human PBMCs and mononuclear cells from mouse spleens and lymph nodes were identified using Viability Dye 506 (Peprotech-BioGems, #62,210-00, New Jersey, USA). Antibodies against surface markers for staining human ILCs included brilliant violet (BV)421-CD56, alexa fluor (AF)647-CD127, phycoerythrin (PE)-CRTH2, BV605-cKit, allophycocyanin-cyanine 7 (APC-Cy7)-CD62L, PE-Cy7-CXCR6, PE-Cy7-CCR6, perCP-Cyanine5.5(PercpCy5.5)-CXCR3, PE-NKp44. Lineage antibodies used are as follows: fluorescein isothiocyanate (FITC)-CD3, FITC-CD4, FITC-CD8, FITC-CD14, FITC-CD15, FITC-CD16, FITC-CD19, FITC-CD20, FITC-CD33, FITC-CD34, FITC-CD203c, FITC-FcεRI, and FITC-CD94. Antibodies against surface markers for staining mouse ILCs included BV605-CD25, APC-α4β7, BV421-Flt3, PE-PD1, PE-Cy7-CD62L, BV711-CXCR6, BV570-CD45, APC-Klrg1, AF750-NKp46, PercpCy5.5-NK1.1, and PE-Cy7-Sca1. Lineage antibodies are as follows: FITC-CD3ε, FITC-CD5, FITC-CD19, FITC-CD45R/B220, FITC-CD11b, FITC-CD11c, FITC-TER-119, FITC-F4/80, FITC-Ly6G/Ly-6C (Gr-1), FITC-CD49b, and FITC- FcεRIα. The gating strategies for identifying human and mouse ILC subsets and ILCPs were based on well-established immunophenotypic criteria [17, 29, 30]. After surface staining, cells were fixed and permeabilized using Transcription Factor Buffer Set (BD Pharmingen, #562,574, New Jersey, USA). Then, cells were stained with the intranuclear antibody PE-RORγt. A minimum of 1 × 10^6^ cells was recorded by LSR Fortessa (BD Biosciences) to be further analyzed by FlowJo 10.8.1. Detailed information for all antibodies is summarized in Supplemental Table 1.

### RNA extraction and quantitative reverse transcriptase PCR (qRT-PCR)

Total RNA was extracted from mouse liver using RNAiso Plus (TAKARA, #108–95-2, Tokyo, Japan) according to the manufacturer’s introduction. The RNA was quantified and then converted to cDNA using Promega GoScript™ Reverse Transcription Mix and Random Primers (Promega, #A2800, Wisconsin, USA). qRT-PCR was performed with Eastep™ qPCR Master Mix (Promega, #LS2068, Wisconsin, USA) using a QuantStudio 5 System (Applied Biosystems, Massachusetts, USA). Gene expression was determined using β-actin as a house-keeping gene, and fold change was calculated using the 2 − ΔΔCT method. qRT-PCR primer sequences are listed in Supplemental Table 2.

### Multifactor detection of cytokines in human serum

The multifactor detection kit (Abclonal, #RK04338, Wuhan, China) was used according to the manufacturer's instructions to detect various pro-inflammatory cytokines in human serum, including IL-23, IL-13, IL-7, IL-22, IL-17, IFN-γ, IL-6, IL-1β, and IL-2. Briefly, 50 μL of standards or samples were successively conjugated with microspheres, antibodies, and fluorescein, and then analyzed using the ABplex multi-indicator flow cytometry co-analysis technology to gain the concentration of each index.

### Statistical analysis

All data are presented as the mean ± SD. Statistically significant differences between dependent groups were made by unpaired two-tailed Student’s *t* test or one-way ANOVA with Tukey’s post hoc honest test. Statistical analyses were performed using GraphPad Prism 9.0 (La Jolla, CA, USA). For multiple comparisons, Bonferroni correction was applied to control for Type I error. Adjusted p values are reported for cytokine and ILC subset analyses. For all comparisons, significance is indicated as *P < 0.05, **P < 0.01, ***P < 0.001, and ****P < 0.0001; ns indicates not significant.

## Results

### Circulating ILCPs and ILC3s are enriched in patients with chronic HBV infection and cirrhosis

**Fig. 1:**
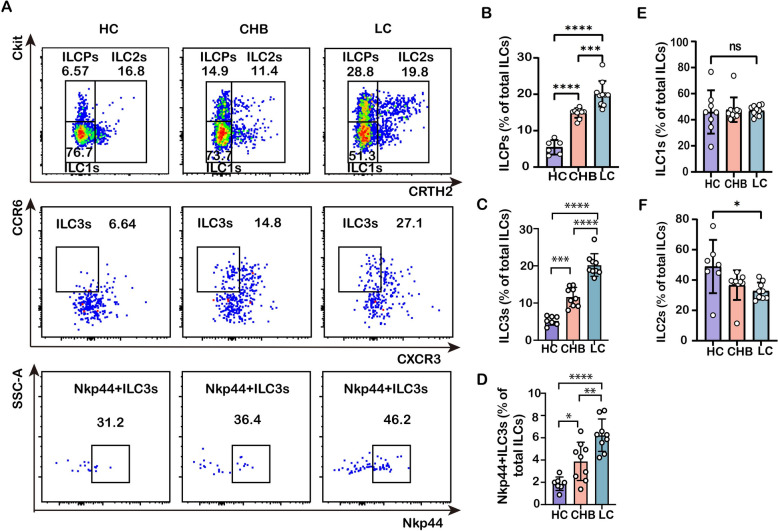
Differential richness of circulating ILCPs and ILC3s in peripheral blood of patients with CHB/LC. **A** The representative flow cytometric staining of ILCPs, ILC1s, ILC2s (upper panel), ILC3s (middle panel), and NKp44^+^ILC3s (lower panel) from HCs, CHB, and cirrhotic patients. **B** The richness of ILCPs in total ILCs was compared between HCs, CHB, and cirrhotic patients. **C** The richness of ILC3 subpopulation in total ILCs was compared between the HCs, CHB, and cirrhotic patients. **D** The richness of NKp44.^+^ILC3s in total ILCs was between the HCs, CHB, and cirrhotic patients. **E** The richness of ILC1s in total ILCs was compared between HCs, CHB, and cirrhotic patients. **F** The richness of ILC2s in total ILCs was compared between HC, CHB, and cirrhotic patients. Statistical significance was determined by one-way ANOVA. Statistical significance is identified by *P < 0.05, **P < 0.01, ***P < 0.001, ****P < 0.0001; “ns” indicates not significant. Each data point represents an individual subject (HCs, *n* = 7; CHB, *n* = 9; LC, *n* = 9)

As an initial step, expression and differentiation of ILCPs from peripheral blood mononuclear cells (PBMCs) were investigated in CHB and cirrhotic patients and healthy volunteers. Flow cytometry was employed to quantitate circulating ILCPs (Lin^−^CD56^−^CD127^+^CRTH2^−^cKit^+^), and subpopulations of ILC1s (Lin^−^CD56^−^CD127^+^CRTH2^−^cKit^−^), ILC2s (Lin^−^CD56^−^CD127^+^CRTH2^+^cKit^+^), and ILC3s (Lin^−^CD56^−^CD127^+^CXCR3^−^CCR6^+^) (Fig. Supplementary 1). The data show that proportions of circulating ILCPs and ILC3 subpopulations were increased in CHB patients compared to healthy individuals, and were even more pronounced in cirrhotic patients (Fig. 1A–C). In contrast, ILC2 subpopulation was shrunk in human HBV infection-associated fibrosis (Fig. 1A/F). Instead, the ILC1 subpopulation was not significantly different in the peripheral circulation between these three groups (Fig. 1A/E). These data suggested that ILCPs were increased and may differentiate toward ILC3 subpopulation in the peripheral circulation with the aggravation of HBV infection-associated fibrosis. It is known that mature ILC3s (NPk44^+^ILC3s in human, NKp46^+^ILC3 in mice) are the main cell type to exert activation and interleukin production [29]; therefore, the richness of NKp44^+^ILC3 subpopulation was further examined in the peripheral circulation, and it was found that the proportion of NKp44^+^ILC3 cells was indeed increased in the CHB and cirrhotic patients compared to the HCs (Fig. 1A/D), suggesting that with the progression of hepatitis B infection-associated fibrosis, ILCPs were further matured to ILC3s, and played an important role in inflammatory response.

### Differential expression of ILCs in the spleen and lymph nodes in mice with HBV infection-associated fibrosis

**Fig. 2:**
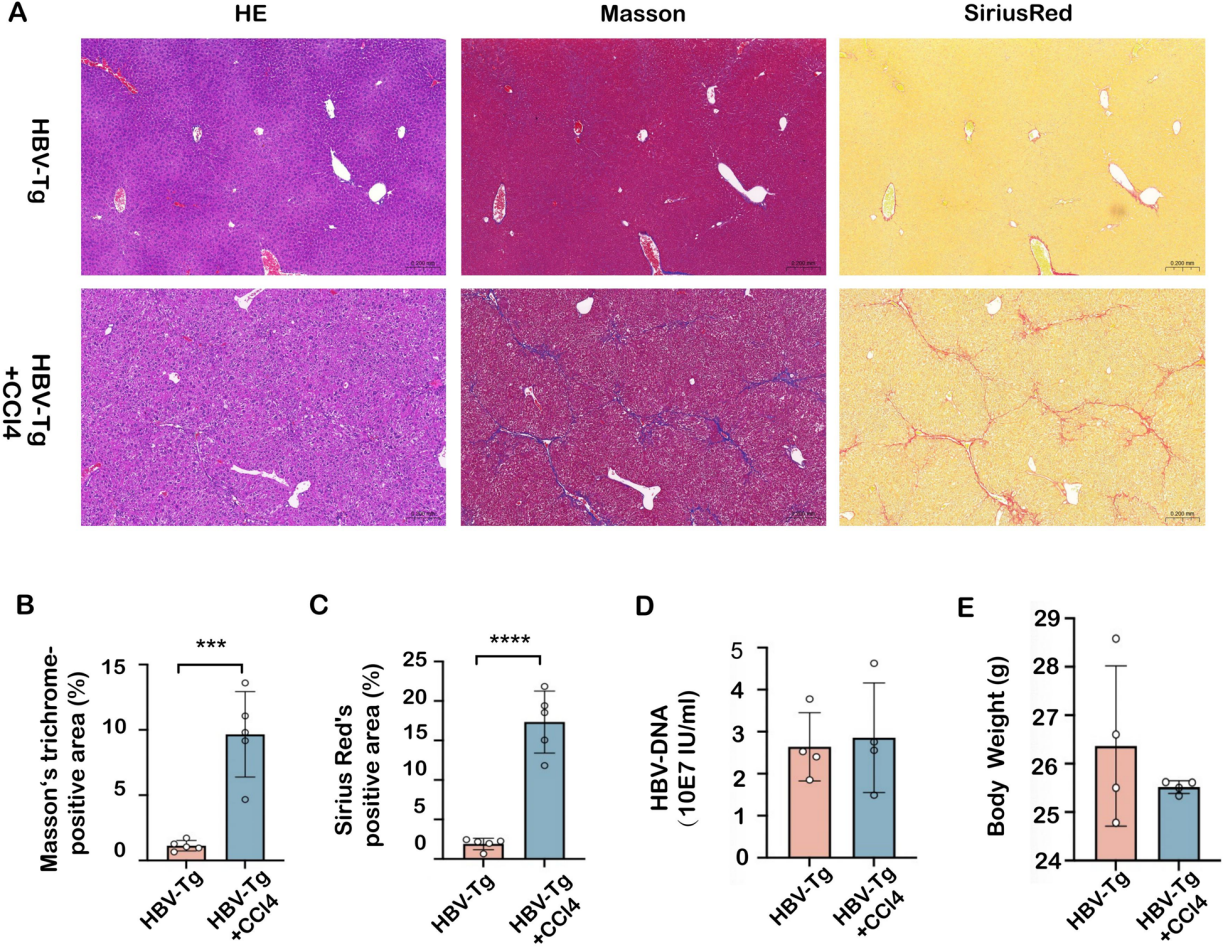
Pathological changes in the mouse liver of HBV infection-associated fibrosis. **A** Liver damage was assessed by H&E staining and fibrosis was shown by Masson and Sirius red staining in liver sections from HBV-Tg and HBV-Tg + CCl_4_-treated groups. Images were taken at original magnification (100x), scale bars = 200 μm. **B**, **C** Statistical graph of Masson-positive and Sirius red-positive areas relative to the whole area. **D** The HBV-DNA quantitative test. **E** The body weight change in the HBV-Tg and HBV-Tg + CCl_4_-treated groups. Statistical significance was determined by unpaired two-tailed Student's *t* test. Statistical significance is identified by *P < 0.05, ***P < 0.001, ****P < 0.0001; ‘‘ns’’ indicates statistically not significant

**Fig. 3:**
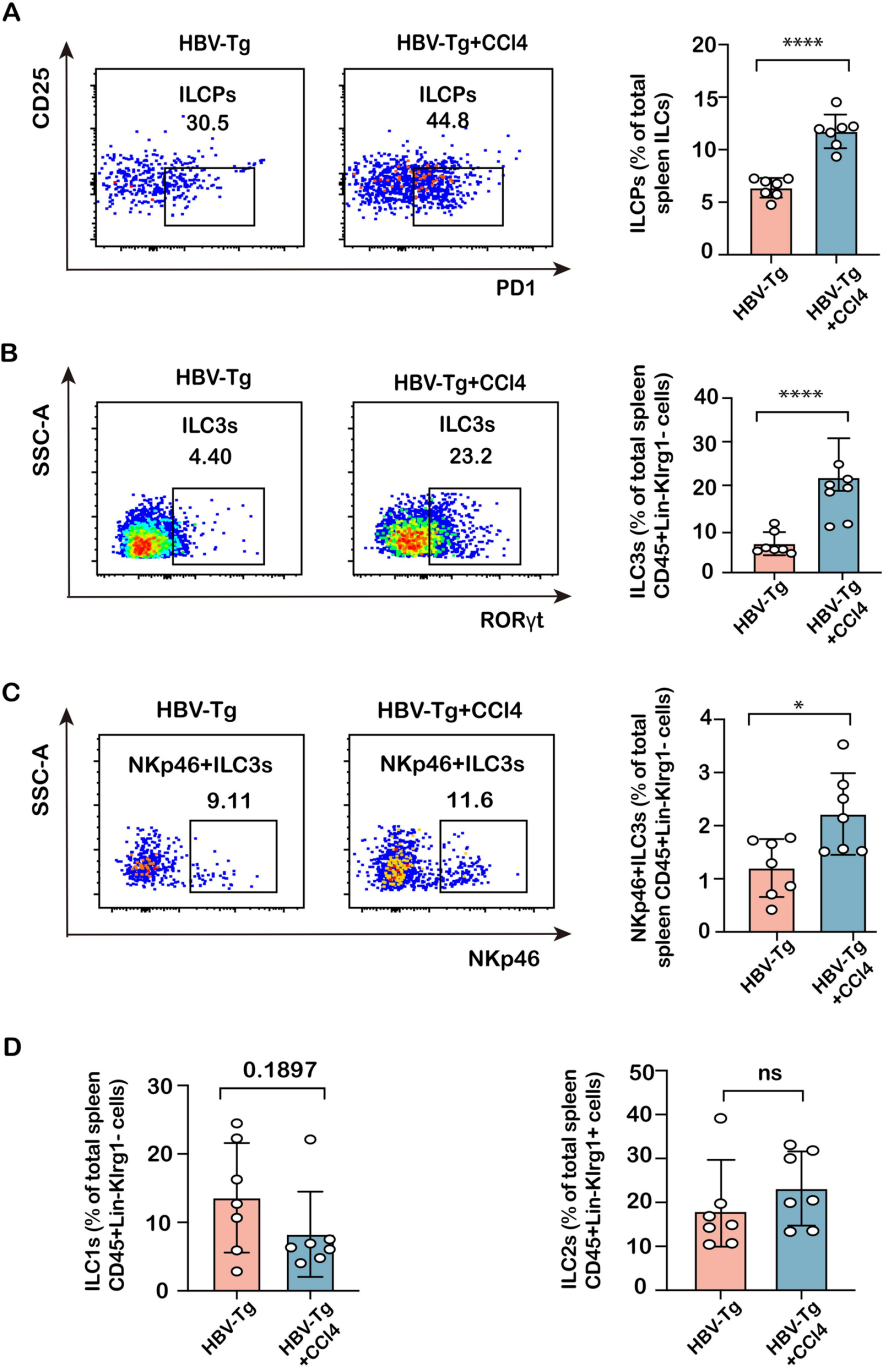
Accumulation of ILC precursors and ILC3s in the spleen of fibrotic HBV-Tg mice. **A** The richness of ILCPs in total spleen ILCs was compared between HBV-Tg and HBV-Tg + CCl_4_-treated groups. **B** The richness of ILC3s in spleen CD45^+^Lin^−^Klrg1^−^ cells was compared between HBV-Tg and HBV-Tg + CCl_4_-treated groups. **C** The richness of NKp46^+^ILC3 was compared between HBV-Tg and HBV-Tg + CCl_4_-treated groups. **D** The richness of splenic CD45^+^Lin^−^Klrg1^−^ILC1 cells and CD45^+^Lin^−^Klrg1^+^ILC2 cells was compared between HBV-Tg and HBV-Tg + CCl_4_-treated groups. Statistical significance was determined by unpaired two-tailed Student's *t* test. Statistical significance is identified by *P < 0.05, ***P < 0.001, ****P < 0.0001; ‘‘ns’’ indicates statistically not significant. The number of mice per group is *n* = 7

**Fig. 4:**
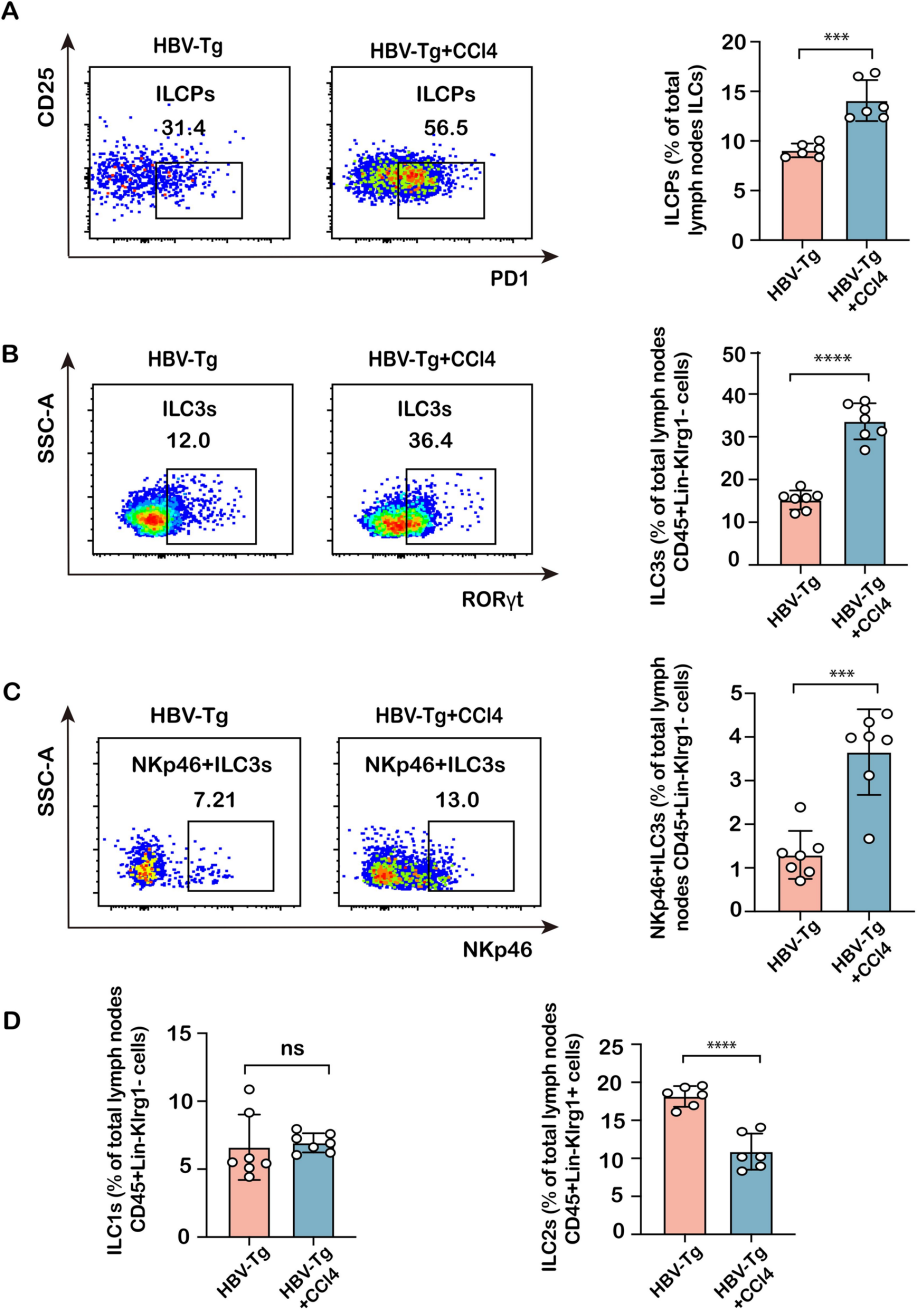
Enrichment of ILC precursors and ILC3s in the lymph nodes of fibrotic HBV-Tg mice. **A** The richness of ILCP fraction in total lymph nodes ILCs was compared between the HBV-Tg and HBV-Tg + CCl_4_-treated groups. **B** The richness of ILC3 subpopulation in lymphoid CD45^+^Lin^−^Klrg1^−^ cells was compared between the HBV-Tg and HBV-Tg + CCl_4_-treated groups. **C** The representative richness of NKp46^+^ILC3 subpopulation in lymphoid CD45^+^Lin^−^Klrg1^−^ cells was compared in the HBV-Tg and HBV-Tg + CCl4-treated groups. **D** The richness of ILC1 subpopulation in lymphoid CD45^+^Lin^−^Klrg1^−^ cells and ILC2 subpopulation in lymphoid CD45^+^Lin^−^Klrg1^+^ cells was compared between the HBV-Tg and HBV-Tg + CCl_4_-treated groups. Statistical significance was determined by unpaired two-tailed Student's *t* test. Statistical significance is identified by *P < 0.05, ***P < 0.001, ****P < 0.0001; ‘‘ns’’ indicates statistically not significant. The number of mice per group is *n* = 7

To further validate the alterations of peripheral ILCPs and ILC3 subpopulation in HBV infection-associated fibrosis, HBV-Tg mice were employed to investigate immune abnormalities with the challenge of CCl_4_ toxicity for 8 weeks to potentiate fibrotic development. As shown in Fig. 2A–C, HBV-Tg + CCl_4_-treated mice caused remarkable collagen accumulation with Sirius red and Masson’s trichrome staining in the liver compared to HBV-Tg mice, representing that CCl_4_ intoxication potentiated fibrotic progression in HBV-Tg mice. There was no significant difference in HBV-DNA replication and body weight between these two groups (Fig. 2D–E). Then, richness of splenic ILCPs (CD127^+^Lin^−^Flt3^−^α4β7^+^CD25^−^PD1^+^), ILC3 (CD45^+^Lin^−^Klrg1^−^RORγt^+^), ILC1 (CD45^+^Lin^−^Klrg1^−^RORγt^−^NK1.1^+^), and ILC2 (CD45^+^Lin^−^Klrg1^+^RORγt^−^NKp46^−^Sca1^+^) subpopulations was determined in ILCs from these mice by flow cytometry (Fig. Supplementary 2). The results revealed that the proportions of ILCPs, ILC3s, and NKp46^+^ILC3s in the spleens of HBV-Tg + CCl_4_-treated mice were significantly higher than those in HBV-Tg mice **(**Fig. 3A–C**)**; whereas ILC1 and ILC2 subpopulations were not different from HBV-Tg mice (Fig. 3D). To define the changes in homing-into-lymph, ILCs were also isolated from mesenteric lymph nodes to verify the proportion of these subpopulations. As shown in Fig. 4, the proportion of ILCPs, ILC3s, and NKp46^+^ILC3s was remarkably increased in ILCs from lymph nodes in HBV-Tg-CCl_4_ + treated mice compared to HBV-transgenic controls (Fig. 4A–C). The proportion of ILC2 subpopulations was downregulated but ILC1 subpopulations were not different (Fig. 4D). These findings suggest that the homing of ILCP subpopulation was increased, and ILCPs were differentiated toward mature ILC3 in mice with hepatitis B infection.

### ILCPs upregulate homing receptors CD62L and CXCR6 in HBV infection-associated fibrosis

**Fig. 5:**
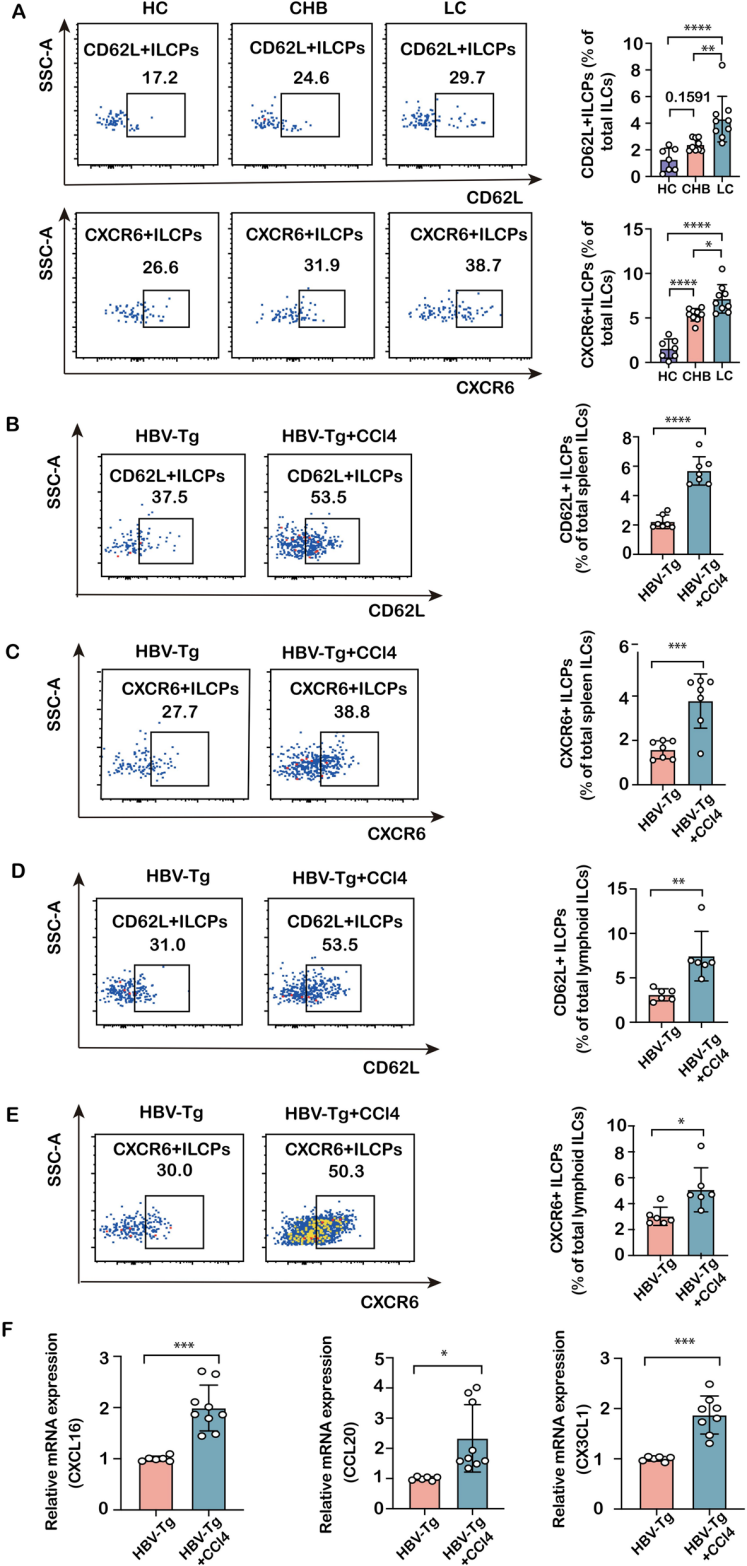
Expression of CD62L and CXCR6 on ILC precursors in human and mouse models of HBV infection-associated fibrosis. **A** Representative flow cytometric staining and the richness of human CD62L^+^ILCPs (upper panel) and CXCR6^+^ILCPs (lower panel) in HCs, CHB and cirrhotic patients. **B** The richness of mouse CD62L^+^ILCP subpopulation in splenic ILCs was compared between HBV-Tg and HBV-Tg + CCl_4_-treated groups. **C** The richness of CXCR6^+^ILCP subpopulation in splenic ILCs was compared between HBV-Tg and HBV-Tg + CCl_4_-treated groups. **D** The richness of CD62L^+^ILCP subpopulation in lymphoid ILCs was compared between HBV-Tg and HBV-Tg + CCl_4_-treated groups. **E** The richness of CXCR6^+^ILCP subpopulation in lymphoid ILCs was compared between the HBV-Tg and HBV-Tg + CCl_4_-treated groups. **F** The mRNA levels of CXCL16, CCL20 and CX3CR1 in mouse liver. Statistical significance was determined by one-way ANOVA (HCs, *n* = 7; CHB, *n* = 9; LC, *n* = 9) and unpaired two-tailed Student's t test in mice (per group is *n* = 7). Statistical significance is identified by *P < 0.05, **P < 0.01, ***P < 0.001, ****P < 0.0001; “ns” indicates not significant

After the confirmation of the homing ILCPs to lymph nodes during HBV infection-associated fibrosis, possible signaling molecules mediating their homing were further investigated. The presence of the receptor CD62L on the surface of ILCPs in human PBMCs demonstrated that the number of CD62L^+^ILCPs was increased in CHB patients compared to healthy subjects, and further increased in cirrhotic patients **(**Fig. 5A). Additionally, an increase in CD62L^+^ILCPs was confirmed in both the spleens and lymph nodes of HBV-Tg mice following CCl_4_ intoxication (Fig. 5B/D), which established substantial evidence in demonstrating the migration of recirculating ILCPs to lymph nodes. Moreover, to confirm the recruitment of circulating ILCPs to the inflamed liver, CXCR6 expression on the surface of ILCPs was examined. The results demonstrated that the proportion of CXCR6^+^ILCPs in both human peripheral blood and mouse spleen or lymph nodes was increased during the progression of hepatitis B infection-associated fibrosis (Fig. 5A/C/E). Correspondingly, mRNA levels of CXCL16, a ligand for CXCR6, CCL20, and CX3CL1, chemokine ligands with strong chemotactic effects on lymphocytes, were markedly increased in the livers of HBV-Tg-CCl_4_-treated mice compared to HBV-Tg mice (Fig. 5F). These data collectively provide evidence for an enhanced presence and ILC3-lineage commitment of ILCPs within lymphoid tissues during the pathogenesis of HBV infection-associated fibrosis.

### The pro-inflammatory cytokine IL-23 correlates with ILCP frequency and homing phenotype

**Fig. 6:**
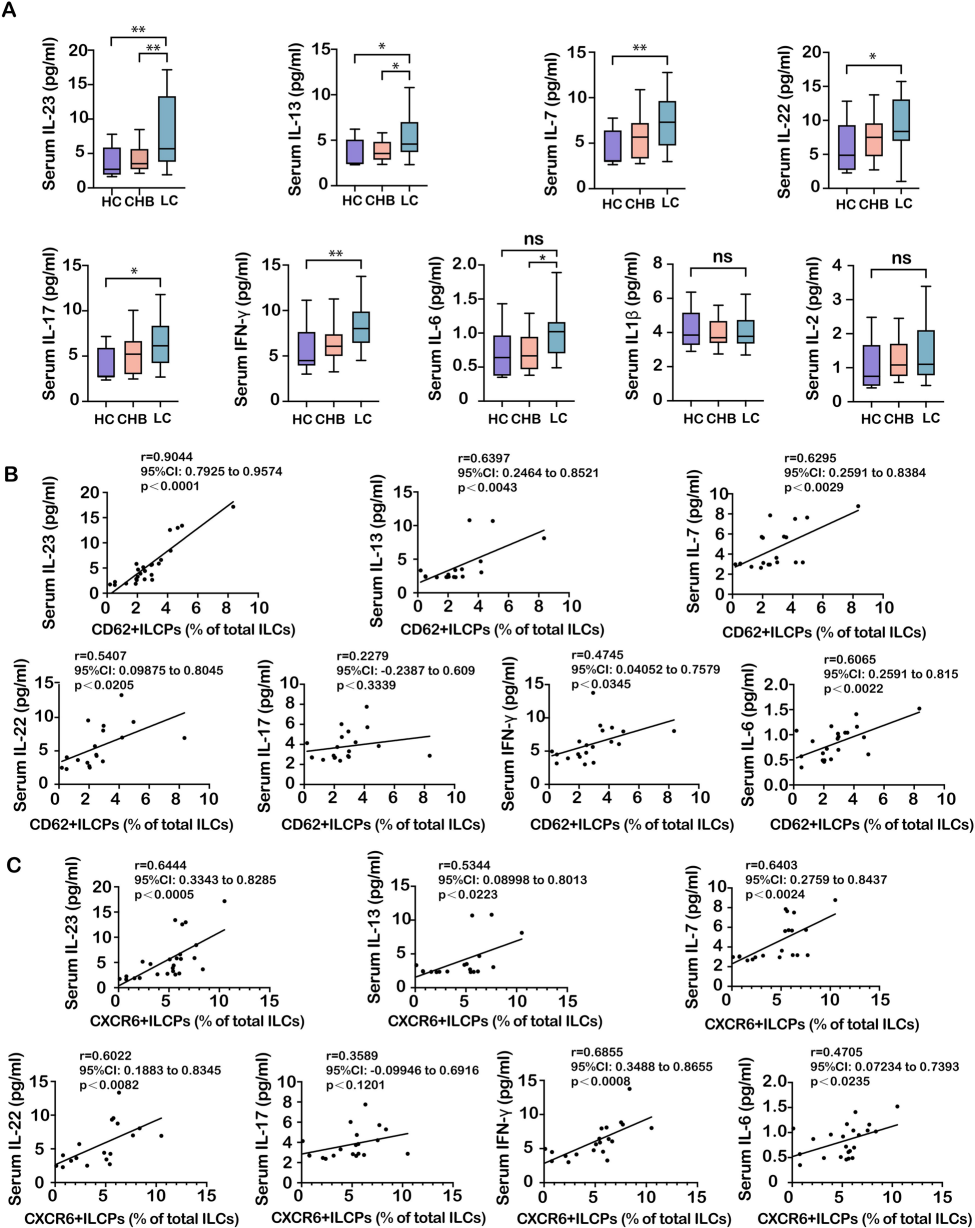
Serum cytokine levels and their correlation with CD62L^+^ and CXCR6^+^ ILCP frequencies. **A** Serum concentrations of IL-23, IL-13, IL-7, IL-22, IL-17, IFN-γ, IL-6, IL-1β, and IL-2 in healthy controls (HCs, *n* = 12), patients with chronic hepatitis B (CHB, *n* = 18), and patients with liver cirrhosis (LC, n = 22). Data were compared by one-way ANOVA. (B, C) Correlation analysis between serum cytokine levels and the frequency of **B** CD62L⁺ ILCPs or **C** CXCR6⁺ ILCPs in the study cohort. Each data point represents an individual subject (HCs, *n* = 12; CHB, n = 18; LC, *n* = 22). The Pearson correlation coefficient (r) and the corresponding *P* value are indicated for each cytokine–subset pair. The solid line represents the linear regression fit, and the shaded area represents the 95% confidence interval. Statistical significance is identified by *P < 0.05, **P < 0.01; “ns” indicates not significant

HBV infection-associated fibrosis is accompanied by increased expression and secretion of several pro-inflammatory cytokines. To determine which cytokines are responsible for the maturation, homing, and recruitment of ILCPs into the liver, a multifactorial assay of human serum pro-inflammatory cytokines was performed in serum of CHB or cirrhotic patients and healthy controls using flow microsphere technology. Compared to HCs, serum levels of IL-23, IL-13, IL-7, IL-22, IL-17, and IFN-γ were elevated in patients with cirrhosis. However, there were no significant differences in the levels of IL-6, IL-1β, and IL-2. Additionally, IL-23, IL-13 and IL-6 levels were higher in the LC group compared to those with chronic CHB (Fig. 6A). The correlation analysis shows that IL-23 has a strong positive correlation with CD62L^+^ILCPs (R2 = 0.8180, p < 0.0001) (Fig. 6B). IL-13, IL-7, and IL-6 also positively correlate with CD62L^+^ILCPs but with lower coefficients than IL-23 (Fig. 6B). Additionally, IL-23, IL-7, IL-22, and IFN-γ have a moderate positive correlation with CXCR6^+^ILCPs (Fig. 6C). No statistically significant correlation was identified between IL-22, IL-17, IFN-γ, and CD62L^+^ ILCPs, nor between IL-13, IL-17, IL-6, and CXCR6^+^ ILCPs. (Fig. 6B, C). In summary, IL-23 appeared to be associated with the homing and recruitment of ILCP cells to the liver and may be involved in the potentiation of HBV infection-associated fibrosis.

### IL-23 directly promotes ILCP differentiation and enhances migratory receptor expression in vitro

**Fig. 7:**
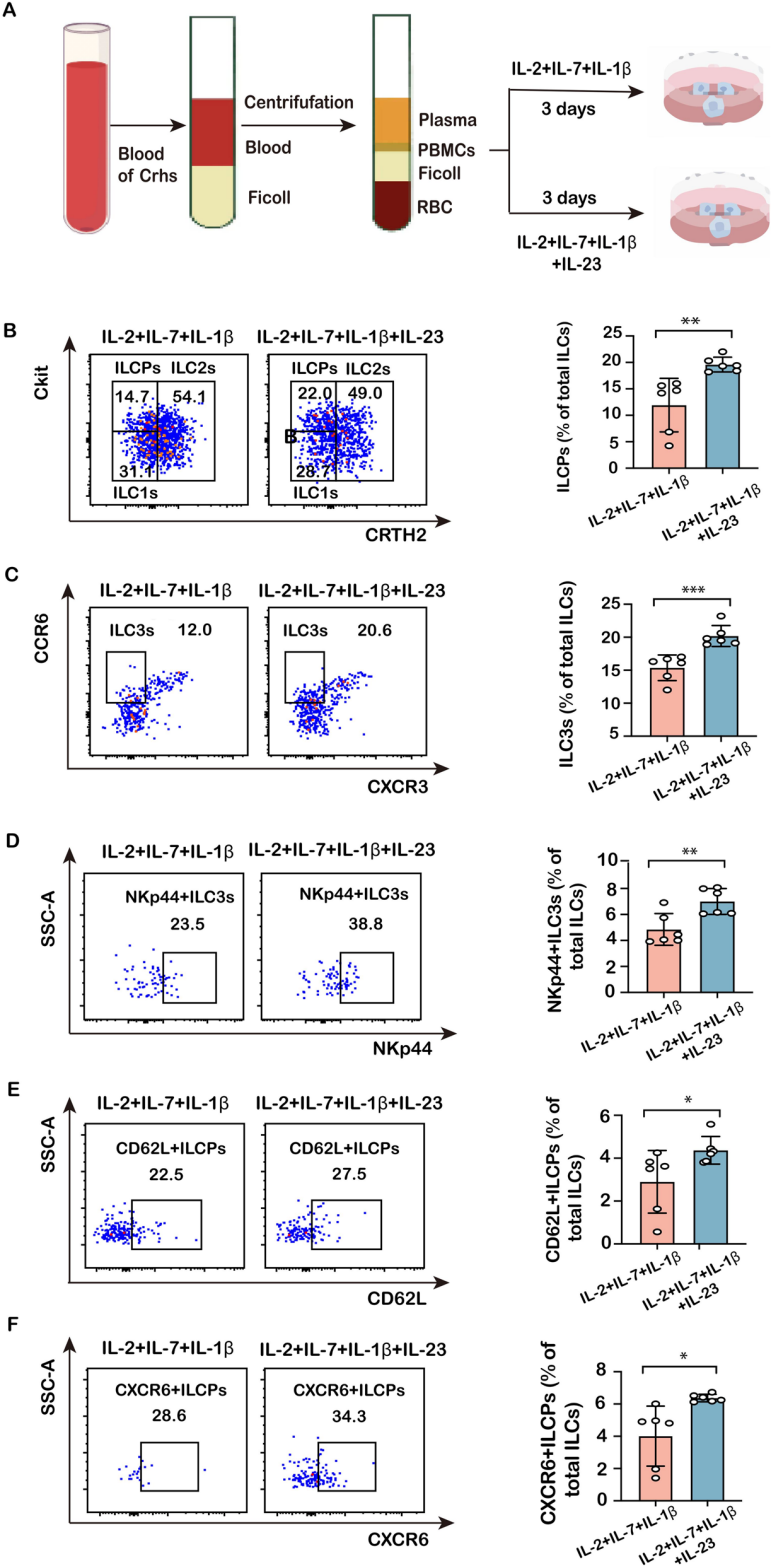
Effect of IL-23 on ILCP and ILC3 frequencies in cultured PBMCs from cirrhotic patients. **A** Schematic diagram of cell treatment. **B** The richness of human ILCP subpopulation in total ILCs was compared between IL-2 + IL-7 + IL1-β and IL-2 + IL-7 + IL1-β + IL-23-treated PBMCs from cirrhotic patients. **C**, **D** The representative flow cytometric staining and frequencies of human ILC3s and human NKp44^+^ILC3 subpopulation in total ILCs were compared in the IL-2 + IL-7 + IL1-β or IL-2 + IL-7 + IL1-β + IL-23 treated PBMC from cirrhotic patients in vitro. **E** The richness of human CD62L^+^ILCPs in total ILCs was compared between IL-2 + IL-7 + IL1-β or IL-2 + IL-7 + IL1-β + IL-23-treated PBMCs from cirrhotic patients **F** The richness of human CXCR6^+^ILCP subpopulation in total ILCs was compared between IL-2 + IL-7 + IL1-treated and IL-2 + IL-7 + IL1-β + IL-23-treated groups. Statistical significance was determined by unpaired two-tailed Student's t test (*n* = 6 biologically independent samples per group). Statistical significance is identified by *P < 0.05, **P < 0.01, ***P < 0.001; “ns” indicates not significant

To further investigate whether IL-23 enhanced the differentiation, homing and recruitment of ILCPs, PBMCs obtained from cirrhotic patients were cultured in the presence of IL-2 + IL7 + IL1β to induce the differentiation toward ILC subpopulations, with or without IL-23 for 3 days (Fig. 7A). Subsequently, the presence of ILC subpopulations was assessed by flow cytometry. The findings demonstrated a significant increase in the richness of ILCP, ILC3, and NKp44^+^ ILC3 subpopulations from PBMCs upon the addition of IL-23 (Fig. 7B–D). Moreover, the presence of CD62L and CXCR6 in ILCP cells was enhanced by the addition of IL-23 (Fig. 7E**/F**). These results suggest that IL-23 may facilitate the homing and recruitment of ILCP cells to the liver and further maturation of ILC3 cells.

### Notch signaling regulates the differentiation and homing phenotype of ILCP

**Fig. 8:**
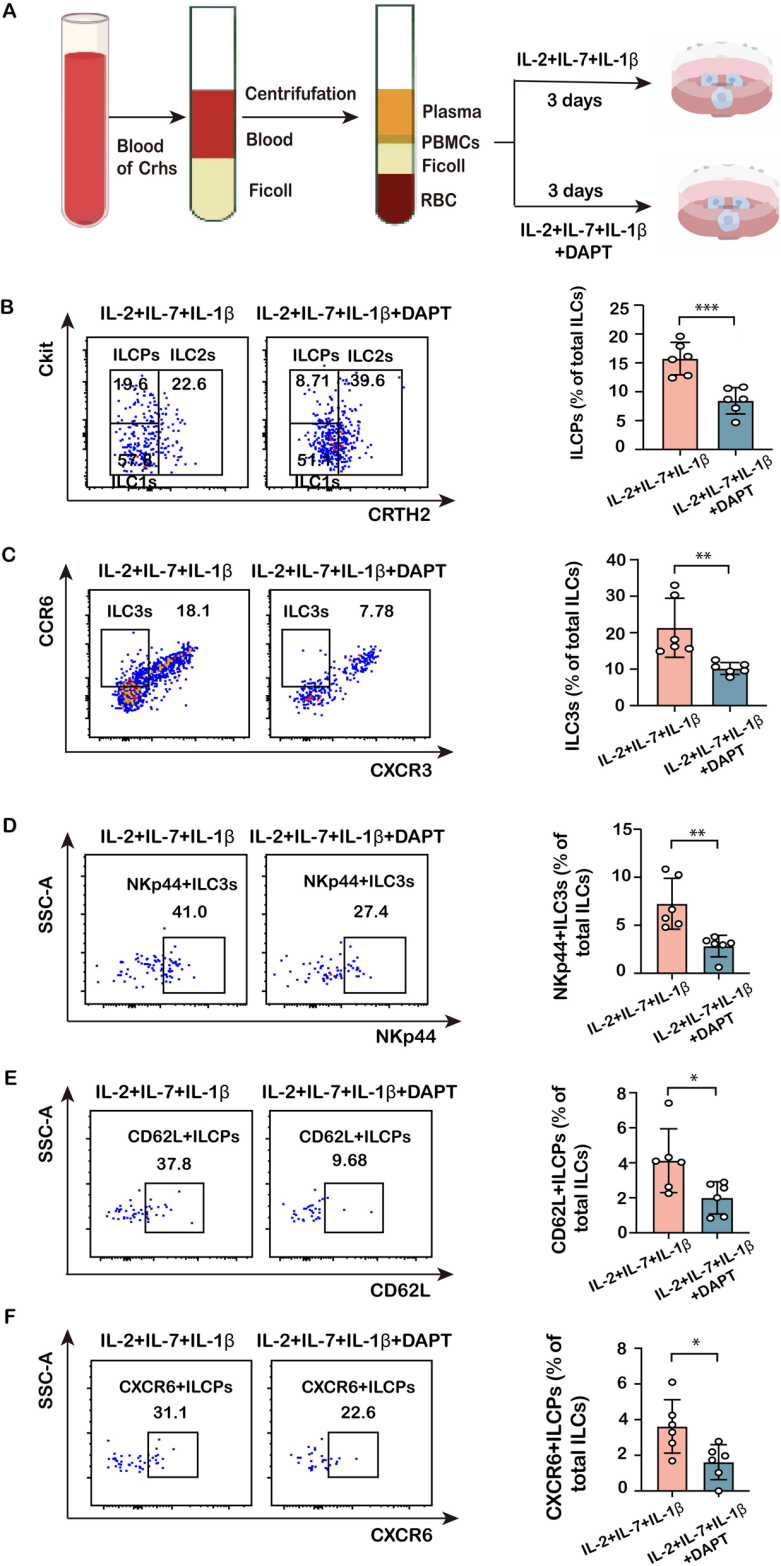
Impact of Notch signaling inhibition on ILC precursor and ILC3 frequencies in vitro. **A** Schematic diagram of cell treatment. **B** The richness of human ILCP subpopulation in total ILCs was compared between IL-2 + IL-7 + IL1-β and IL-2 + IL-7 + IL1-β + DAPT-treated PBMCs from cirrhotic patients. **C**, **D** The richness of human ILC3 subpopulation and human NKp44 + ILC3 subpopulation in total ILCs was compared between IL-2 + IL-7 + IL1-β and IL-2 + IL-7 + IL1-β + DAPT-treated PBMCs from cirrhotic patients. **E** The richness of human CD62L + ILCP subpopulation in total ILCs was compared between IL-2 + IL-7 + IL1-β and IL-2 + IL-7 + IL1-β + DAPT-treated PBMCs from cirrhotic patients. **F** The richness of human CXCR6 + ILCP subpopulation in total ILCs was compared between IL-2 + IL-7 + IL1-β and IL-2 + IL-7 + IL1-β + DAPT-treated PBMCs from cirrhotic patients. Statistical significance was determined by unpaired two-tailed Student's *t* test (*n* = 6 biologically independent samples per group). Statistical significance is identified by *P < 0.05, **P < 0.01, ***P < 0.001; “ns” indicates not significant

Notch is an important signaling pathway in both ILC cell fate decision and ILC immune response. Thus, PBMCs of cirrhotic patients were cultured for 3 days in the presence or absence of a Notch receptor inhibitor, DAPT (Fig. 8A). The flow cytometric results suggested that the richness of ILCP, ILC3, and NKp44^+^ILC3 cells was significantly decreased by the addition of DAPT (Fig. 8B–D), suggesting that the inhibition of Notch signaling suppressed the differentiation of ILCPs to ILC3 subset cells and hampered the maturation of the latter. Moreover, DAPT reduced the proportions of CD62L^+^ILCP and CXCR6^+^ILCP cells (Fig. 8E/F), indicating that inhibition of Notch receptor hindered the homing and recruitment of ILCP cells. These findings provide further support that Notch signaling contributes to human ILCP differentiation and the maintenance of their homing phenotype, aligning with prior studies [21, 30].

## Discussion

This study provides compelling evidence for the expansion and phenotypic alteration of circulating innate lymphoid cell precursors (ILCPs) during the progression of fibrosis in chronic HBV infection. We demonstrate that ILCPs, particularly those expressing the homing receptors CD62L and CXCR6, are increased in the peripheral blood of patients with CHB and cirrhosis, as well as in the secondary lymphoid organs of HBV-transgenic mice with exacerbated fibrosis. This expansion was accompanied by a concomitant rise in mature ILC3s, suggesting a coordinated differentiation pathway from precursors to effector cells within the chronic liver disease milieu. Furthermore, our in vitro functional assays establish that the cytokine IL-23 and the Notch signaling pathway are potent drivers of ILCP differentiation into ILC3s and the upregulation of these critical homing receptors.

The observed preferential expansion of the ILC3 subset in both our clinical cohorts and murine model aligns with and extends previous reports underscoring the role of ILC3s in liver fibrosis of various etiologies [15, 16, 31]. However, whereas prior studies, including our own, primarily focused on the effector functions of mature, tissue-resident ILC3s [14], the present work pivots the focus toward their circulating precursors. This reframes the understanding of ILC biology in liver disease, proposing that the intrahepatic pool of ILC3s may be dynamically replenished by recruited circulating ILCPs. This paradigm of systemic precursor recruitment to peripheral tissues is conceptually supported by foundational work in other organ systems [17, 32]. The significant enrichment of CD62L⁺ and CXCR6⁺ ILCPs substantiates this notion, as these receptors are mechanistically implicated in lymphocyte entry into lymphoid organs and retention within inflamed tissues, respectively [18, 19, 33]. The concurrent upregulation of the CXCR6 ligand, CXCL16, in fibrotic murine livers provides a plausible chemotactic mechanism for the recruitment of CXCR6-expressing ILCPs, a pathway also implicated in the trafficking of other lymphocyte populations to the damaged liver [34, 35].

The implicated roles of IL-23 and Notch signaling in regulating ILCPs constitute another significant finding. The strong correlation between serum IL-23 levels and CD62L⁺ ILCP frequency, combined with the in vitro capacity of IL-23 to promote both ILCP differentiation and homing receptor expression, nominates IL-23 as a key upstream regulator in this process. This is consistent with the well-documented role of IL-23 in orchestrating IL-17-producing cell populations, including Th17 cells and ILC3s, in chronic inflammatory and autoimmune settings [36, 37], and its reported elevation in the context of chronic HBV infection [38]. Similarly, the suppression of ILCP differentiation and homing receptor expression upon Notch pathway inhibition with DAPT resonates with extensive literature identifying Notch as a critical arbiter of ILC fate and function across mouse and human systems [21, 22, 39]. The specific role of Notch in promoting ILC3 differentiation from human hematopoietic progenitors has been particularly well elucidated [40, 41].

Several limitations warrant consideration when interpreting our findings. First, the enrolled patient population was uniformly on long-term antiviral therapy with sustained viral suppression. Consequently, the observed immune alterations are more directly associated with the established stage of fibrosis and/or persistent antigenic exposure, rather than active viral replication, a clinically prevalent scenario that merits specific immunological scrutiny [42]. Second, the absence of a non-HBV liver disease control group (e.g., NASH or alcohol-related cirrhosis) precludes definitive conclusions regarding the specificity of the described ILCP/ILC3 signature to HBV etiology; it may represent a common immunopathological pathway in advanced liver fibrosis, as hinted by studies in other chronic liver diseases [31, 43]. Third, the utilization of the CCl₄-accelerated HBV-transgenic model implies that fibrosis is predominantly driven by repetitive toxic injury. While this model is a valuable and widely used tool for studying fibrosis in the context of HBV antigen persistence [24], it does not fully recapitulate the immune-mediated fibrogenesis central to human HBV infection. Finally, the proposed homing of ILCPs to the liver and the precise in vivo contributions of IL-23 and Notch signaling are inferred from correlative and in vitro data; direct validation through future studies employing intrahepatic immune cell profiling, adoptive cell transfer and tracking, and in vivo genetic or pharmacological pathway modulation is required.

Notwithstanding these limitations, our findings establish a foundational link between circulating ILCPs and the progression of HBV-related fibrosis. The identification of an expanded, migration-competent ILCP population unveils new avenues for therapeutic immunomodulation. Strategies aimed at intercepting the recruitment (e.g., via CXCR6 blockade) or differentiation (e.g., via IL-23 or Notch inhibition) of pro-fibrotic ILC lineages at the precursor level could potentially evolve into novel approaches to mitigate liver fibrosis in chronic HBV infection and potentially other chronic liver diseases [43].

## Conclusion

In summary, our investigation demonstrates that circulating ILC precursors are expanded and exhibit an enhanced migratory phenotype during the progression of HBV infection-associated liver fibrosis. These ILCPs display a differentiation bias toward the ILC3 lineage, a process that can be potently facilitated by the IL-23 cytokine and the Notch signaling pathway in vitro. While these findings illuminate a previously under-explored dimension of innate immunity in chronic liver disease, they concurrently underscore the imperative for further research to establish direct causal mechanisms in vivo and to delineate the etiological specificity of these observations. A deeper understanding of the ILC life cycle, spanning from circulating precursor to tissue-resident effector, holds promise for yielding novel immunotherapeutic targets aimed at arresting fibrotic progression.

## Supplementary Information

Supplementary material 1.

## Data Availability

Files, are available from the corresponding author upon reasonable request.

## Abbreviations

CXCR6: CXC chemokine receptor 6
DMSO: Dimethyl sulfoxide
DMEM: Dulbecco's modified Eagle's medium
DAPT: N-[N-(3,5-difluorophenacetyl)-l-alanyl]-S-phenylglycine t-butyl ester
FBS: Fetal bovine serum
HBV: Hepatitis B virus
H&E: Hematoxylin and eosin
ILCs: Innate lymphoid cells
ILCPs: ILC precursors
IL17A: Interleukin-17A
IL22: Interleukin-22
IL23: Interleukin-23
PBS: Phosphate buffered saline
qRT-PCR: Quantitative reverse transcriptase polymerase chain reaction
RPMI: Roswell Park Memorial Institute
Th1: T helper 1
Th2: T helper 2
Th17: T helper 17
