# Supplementary material for: Proliferation, migration, and differentiation of circulating ILC precursors in HBV infection-associated fibrosis
Source: Eur J Med Res. 2026 Jan 5;31:196. doi: 10.1186/s40001-025-03803-w (PMC12870510; doi:10.1186/s40001-025-03803-w)
Supplement: Supplementary file 1 — Supplementary material 1. [file 40001_2025_3803_MOESM1_ESM.docx]

**Supplemental Tables**

**Supplemental Table 1 Antibody list**

| Antibodies | Source | Catalog | Clone |
| --- | --- | --- | --- |
| Brilliant Violet 421TM anti-human CD56 | BioLegend | 362552 | 5.1H11 |
| Alexa Fluor® 647 anti-human CD127 | BioLegend | 351318 | A019D5 |
| PE anti-human CD294 (CRTH2) | BioLegend | 350106 | BM16 |
| Brilliant Violet 605™ anti-human CD117 (c-kit) | BioLegend | 313218 | 104D2 |
| APC/Cyanine7 anti-human CD62L | BioLegend | 304814 | DREG-56 |
| PE/Cyanine7 anti-human CD186 (CXCR6) | BioLegend | 356012 | K041E5 |
| PE/Cyanine7 anti-human CD196 (CCR6)  PerCP/Cyanine5.5 anti-human CD183 (CXCR3)  PE anti-human CD336 (NKp44)  FITC anti-human CD3  FITC anti-human CD4 | BioLegend  BioLegend  BioLegend  BioLegend  BioLegend | 353418  353714  325107  317306  357406 | G034E3  G025H7  P44-8  OKT3  A161A1 |
| FITC anti-human CD8 | BioLegend | 344704 | SK1 |
| FITC anti-human CD14 | BioLegend | 301804 | M5E2 |
| FITC anti-human CD15 (SSEA-1) | BioLegend | 301904 | HI98 |
| FITC anti-human CD16 | BioLegend | 302006 | 3G8 |
| FITC anti-human CD19 | BioLegend | 363008 | SJ25C1 |
| FITC anti-human CD20 | BioLegend | 302304 | 2H7 |
| FITC anti-human CD33 | BioLegend | 303304 | HIM3-4 |
| FITC anti-human CD34 | BioLegend | 343604 | 561 |
| FITC anti-human CD203c (E-NPP3) | BioLegend | 324614 | NP4D6 |
| FITC anti-human CD94  FITC anti-mouse CD3ε  FITC anti-mouse CD5  FITC anti-mouse CD19  FITC anti-mouse/human CD45R/B220  FITC anti-mouse/human CD11b  FITC anti-mouse CD11c  FITC anti-mouse TER-119  FITC anti-mouse F4/80  FITC anti-mouse Ly-6G/Ly-6C (Gr-1)  FITC anti-mouse CD49b  FITC anti-mouse FcεRIα  Brilliant Violet 605™ anti-mouse CD25  APC anti-mouse α4β7  Brilliant Violet 421™ anti-mouse CD135 (Flt3)  PE anti-mouse CD279 (PD-1)  PE/Cyanine7 anti-mouse CD62L  Brilliant Violet 711™ anti-mouse CD186 (CXCR6)  Brilliant Violet 570™ anti-mouse CD45  APC anti-mouse Klrg1  APC/Fire^TM^ 750 anti-mouse CD335 (Nkp46)  PerCP-Cy™5.5 anti-Mouse NK-1.1  PE-Cy7 Rat anti-mouse Ly-6A/E(Sca1) | BioLegend  BioLegend  BioLegend  BioLegend  BioLegend  BioLegend  BioLegend  BioLegend  BioLegend  BioLegend  BioLegend  BioLegend  BioLegend  BioLegend  BioLegend  BD Pharmingen  BioLegend  BioLegend  BioLegend  BD Pharmingen  BioLegend  BD Pharmingen  BD Pharmingen | 305504  100305  100605  115505  103205  101205  117305  116205  123107  108405  103503  134305  102035  120607  135315  551892  104418  151111  103136  561620  137632  551114  558162 | DX22  145-2C11  53-7.3  6D5  RA3-6B2  M1/70  N418  TER-119  BM8  RB6-8C5  HMα2  MAR-1  PC61  DATK32  A2F10  J43  MEL-14  SA051D1  30-F11  2F1  29A1.4  PK136  D7 |

**Supplemental Table 2** **Primer sequences**

| Gene | Forward Primer（5’-3’） | Reverse Primer（5’-3’） |
| --- | --- | --- |
| *CXCL16* | CCTTGTCTCTTGCGTTCTTCC | TCCAAAGTACCCTGCGGTATC |
| *CCL20*  *CX3CL1* | GCCTCTCGTACATACAGACGC  ACGAAATGCGAAATCATGTGC | CCAGTTCTGCTTTGGATCAGC  CTGTGTCGTCTCCAGGACAA |
| *β-Actin* | GGCTGTATTCCCCTCCATCG | CCAGTTGGTAACAATGCCATGT |

**Supplemental Figures**

**
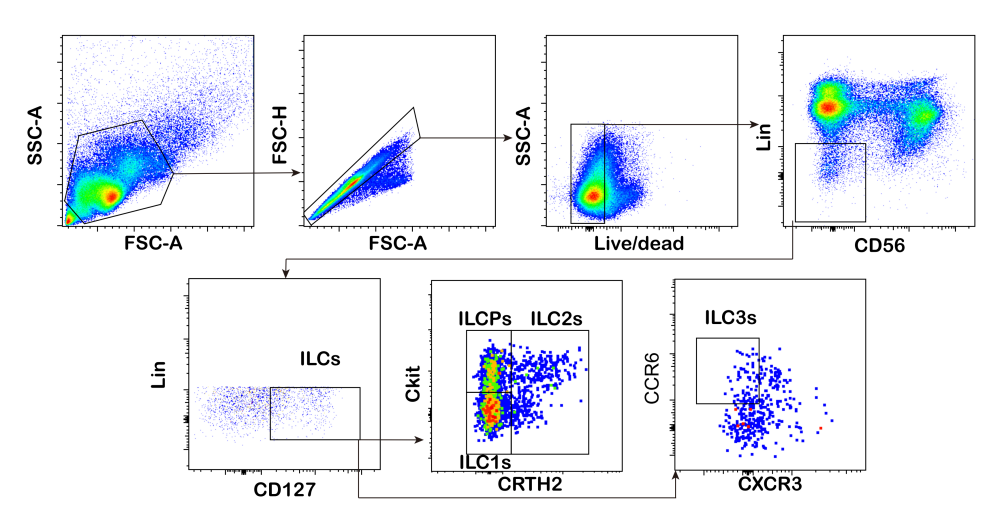
**

**Supplemental Fig.1 The gating strategies of human peripheral ILCPs, ILC1s, ILC2s and ILC3s.** Flow cytometry was employed to quantitate circulating ILCPs (Lin^-^CD56^-^CD127^+^CRTH2^-^cKit^+^), and subpopulations of ILC1s (Lin-CD56^-^CD127^+^CRTH2^-^cKit^-^), ILC2s (Lin^-^CD56^-^CD127^+^CRTH2^+^cKit^+^) and ILC3s (Lin^-^CD56^-^CD127^+^CXCR3^-^CCR6^+^)


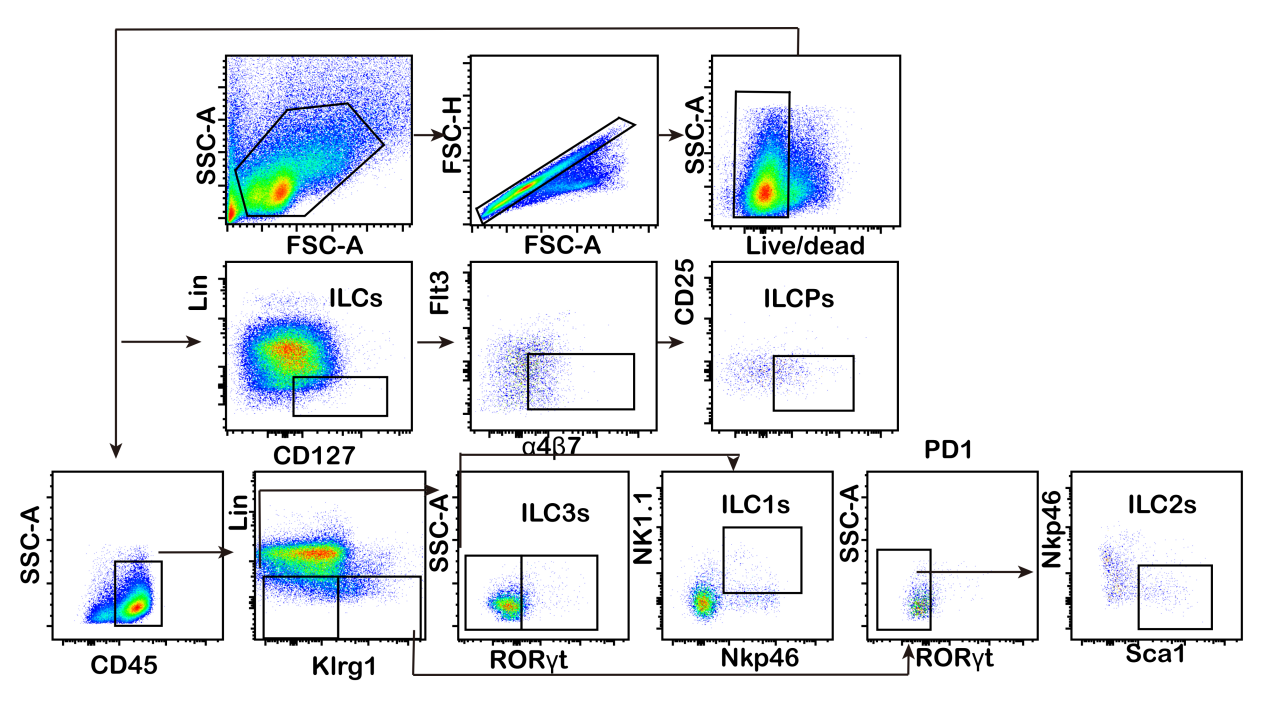


**Supplemental Fig.2 The gating strategies of mouse ILCPs, ILC1s, ILC2s and ILC3s.** Flow cytometric detection of mouse ILCPs (CD127^+^Lin^-^Flt3^-^α4β7^+^CD25^-^PD1^+^), ILC3 (CD45^+^Lin^-^Klrg1^-^RORγt^+^), ILC1 (CD45^+^Lin^-^Klrg1^-^RORγt^-^NK1.1^+^), and ILC2 (CD45^+^Lin^-^Klrg1^+^RORγt^-^NKp46^-^Sca1^+^)
